# Ribosomal protein L5 (RPL5)/ E2F transcription factor 1 (E2F1) signaling suppresses breast cancer progression via regulating endoplasmic reticulum stress and autophagy

**DOI:** 10.1080/21655979.2022.2052672

**Published:** 2022-03-16

**Authors:** Xiaoping Ma, Yan Li, Bing Zhao

**Affiliations:** Breast Internal Medicine Department, The 3rd Affiliated Teaching Hospital of XinJiang Medical University(Affiliated Tumor Hospital), Urumqi, China

**Keywords:** Breast cancer, endoplasmic reticulum stress, autophagy, RPL5, E2F1

## Abstract

Endoplasmic reticulum stress (ERS) is associated with breast cancer progression. However, the potential role of ribosomal protein L5 (RPL5) on ERS in breast cancer remains unclear. This study aimed to determine the role of RPL5/E2F transcription factor 1 (E2F1) in breast cancer. It was found that RPL5 was downregulated in breast cancer cells and tissues. Additionally, overexpression of RPL5 inhibited cell proliferation. Moreover, the levels of ERS and autophagy markers were estimated using western blotting. Overexpression of RPL5 induced ERS and suppressed autophagy. Additionally, RPL5 downregulated E2F1, which was overexpressed in breast cancer cells. However, E2F1 knockdown promoted the transcriptional activation of glucose regulated protein 78 (GRP78), suppressed ERS response, and promoted autophagy. Rescue assays indicated that the effects of RPL5 on ERS and autophagy were abolished by E2F1. Taken together, RPL5 inhibited the growth of breast cancer cells by modulating ERS and autophagy via the regulation of E2F1. These findings suggest that RPL5 has a tumor-suppressive effect in breast cancer.

## Graphical abstract

**Figure:**
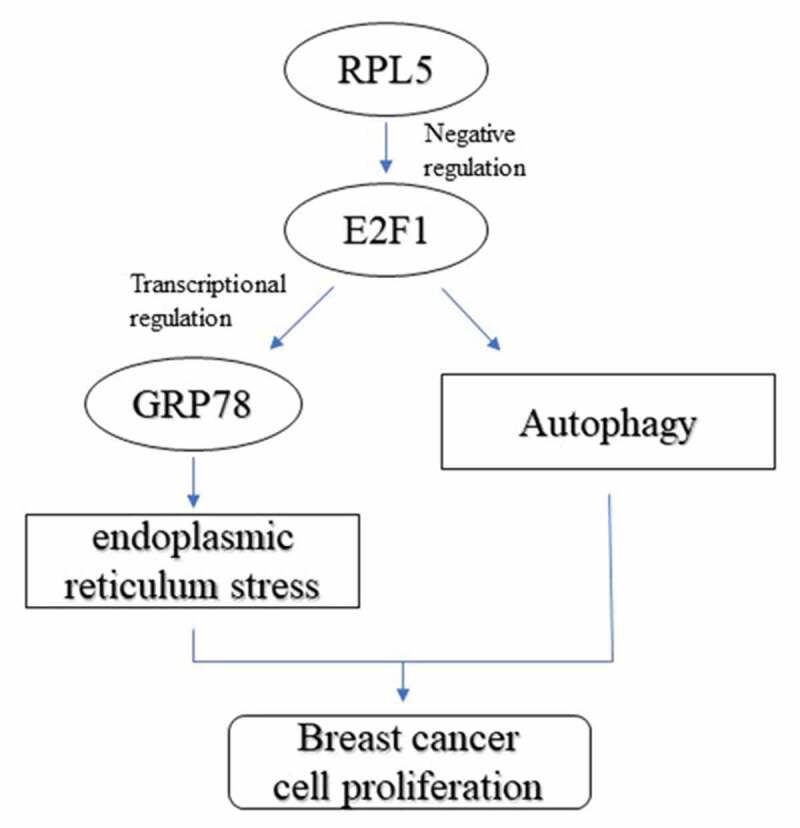

## Introduction

Breast cancer is the most malignancy among women worldwide. The mortality rate of breast cancer has decreased since the 1990s [1]. Recently, screening, diagnosis, and treatment of breast cancer have improved, with an overall survival rate of 90% [2,3]. However, owing to tumor metastasis and drug resistance, the overall survival rate of patients at advanced stages remains poor [^3–5^].

The endoplasmic reticulum (ER) is a crucial organelle for the synthesis and folding of new transmembrane and secreted proteins. ER homeostasis is vital for natural cellular activity [6]. ER stress (ERS) induces the accumulation of large amounts of misfolded proteins in the ER, thereby activating the unfolded protein response (UPR) to clear misfolded proteins [7]. When ERS continues to exist, the damaged ER can be engulfed and degraded by autophagy vesicles. Degraded fragments of the ER can be reassembled into new ones [8]. Chronic ERS and UPR deficiency are the main causes of diseases, including malignancy [9]. Tumor cells are exposed to an environment that alters protein homeostasis in the ER, resulting in ERS [10]. Abnormal activation of ERS regulates tumor growth, metastasis, and drug resistance [11]. Therefore, the induction of ERS may be a potential anticancer treatment strategy.

Ribosomal protein L5 (RPL5), a member of the ribosomal protein (RP) family, belongs to the 60s ribosomal subunit and acts as a sensor of ribosomal stress [12]. Because the interaction between RPL5 and Hdm2 is promoted by UPR induction, ribosomal stress is associated with ERS [13]. It has been indicated that RP may trigger the development of cancers, including endometrial cancer, acute leukemia, colorectal cancer, and glioma [14]. Downregulation of RPL5 may contribute to poor prognosis, suggesting that RPL5 may be a diagnostic biomarker for human cancers [15,16]. In breast cancer, downregulation of RPL5 accelerates the development of tumors and leads to poor prognosis [17]; however, the effects of RPL5 on ERS in breast cancer remain unknown.

In this study, we aimed to explore the potential role of RPL5 in breast cancer and its underlying mechanisms. We hypothesized that RPL5 regulated the progression of breast cancer via regulating ERS and autophagy. Mechanistically, the regulatory function of RPL5 was exerted by modulating E2F transcription factor 1 (E2F1). The study will pvovide a novel insight for breast cancer therapy.

## Materials and methods

### Tissue collection

Clinical specimens (n = 30) were collected from patients with breast cancer at The Third Affiliated Teaching Hospital of XinJiang Medical University (Affiliated Tumor Hospital). Patients received anticancer therapy were excluded in this study. After surgery, the tissues were immediately stored at −80°C. The study protocol was approved by the Ethics Committee of The Third Affiliated Teaching Hospital of XinJiang Medical University (Affiliated Tumor Hospital) prior to the study (Ethical Application ID: [2019]04–293-01). All the participants provided informed consent. Correlation between RPL5 and clinical characteristics is shown in Table 1.

**Table 1.** Correlation between RPL5 expression and clinical characteristics

Factors			RPL5 expression		P-value
			Low	High	
Ages (years)	≤50	22	10	12	0.1514
	>50	8	6	2	
TNM stage	I	4	1	3	0.0322
	II	18	7	10	
	III	9	8	1	
	IV	0	0	0	
Tumor size (cm)	<3	13	6	7	0.0371
	≥3	17	14	3	
Lymph node metastasis	Negative	13	6	7	0.0303
	Positive	17	9	8	

### Immunohistochemical (IHC) analysis

IHC assay was performed according to the previously described [18]. Paraffin sections were dewaxed and rehydrated. Endogenous peroxidase activity was eliminated using 0.3% H_2_O_2_ for 15 min. The blocking was performed using normal goat serum. Then, the slides were incubated with anti-RPL5 or anti-E2F1 at 4°C overnight and incubated with goat anti-rabbit IgG at 37°C for 30 min. After washing with PBS, the sliders were stained with DAB solution and counterstained with hematoxylin.

### Cell culture

The breast cancer cell line MCF-7 and normal breast epithelial cell line MCF-10A were obtained from the American Type Culture Collection (ATCC). The cells were incubated in DuIbecco’s modified eagIe’s medium (DMEM) containing 10% fetal bovine serum (FBS) and 1% penicillin/streptomycin at 37°C with 5% CO_2_. Autophagy inhibitor 3-MA (10 mmol/L; Sigma-Aldrich) was used as the positive control.

### Cell transfection

RPL5 overexpressing vector, E2F1 overexpressing vector, E2F1-specific siRNAs, empty vector, and siRNA were purchased from GenePharm, Shanghai. MCF-7 cells were seeded into 6-well plates and transfection was conducted with Lipofectamine 3000 (Invitrogen) following the manufacturer’s protocol for 48 h.

### Cell counting kit-8 (CCK-8) assay

Transfected cells were seeded into 96-well plates at the concentration of 1х 10^3^ cells/well and cultured at 37°C for 0, 12, 24, and 48 h, respectively. The cells were then treated with 10 µL CCK-8 reagent and cultured at 37°C for 2 h. The absorbance at 450 nm was measured using a microplate reader.

### 5-ethynyl-20-deoxyuridine (EdU) assay

Click-iT EdU Imaging Kit (Invitrogen) was used to analyze tumor cell growth according to manufacturer’s instrument [19]. The cells were fixed with 4% paraformaldehyde for 30 min, permeated with Triton X-100, and stained with EdU dye. DAPI was used to bind the DNA. The cells were photographed using a fluorescence microscope (Olympus).

### Real-time quantitative PCR (qPCR)

Total RNA was extracted from tissues and cells using TRizol reagent (Invitrogen). Reverse transcription was performed using the PrimeCcript RT Master Mix (Takara). qPCR was then performed using FastStart™ PCR Master (Roche). The relative expression of RPL5 and E2F1 (fold-change) was calculated using the 2^−ΔΔCt^ method, with GAPDH as the housekeeping control.

### Western blotting

Protein was extracted from the MCF-7 cells, and the total protein concentration was estimated using a BCA kit. Next, the protein was separated using 12% SDS-PAGE and transferred onto PVDF membranes. After blocking with 5% skim milk for 1 h, the membranes were incubated with primary antibodies at 4°C overnight. The next day, the membranes were incubated with secondary antibodies. The bands were captured using ECL Western Blotting Substrate (Solarbio).

### Bioinformatics

The expression of RPL5 and E2F1 was analyzed using StarBase database. The survival rate related to RPL5 was acquired from Kaplan online database. The DNA motif of E2F1 and potential binding sites between E2F1 and GRP78 were predicted using the JASPAR online tool.

### Dual-luciferase reporter assay

The GRP78 promoter sequence was cloned into the pGL3 vector. Mutant sequences of the GRP78 promoter were also cloned into the pGL3 vector. Cells were co-transfected with WT or MUT GRP78 and siRNA or si-E2F1 by using Lipofectamine 3000 for 48 h. The luciferase activity was measured using a luciferase kit (Promega). *Renilla* luciferase activity was used as the internal control.

### Chromatin Immunoprecipitation (ChIP) assay

CHIP analysis was performed according to a previous study [20]. The cells were lysed in SDS lysis buffer. The lysates were incubated with anti-IgG and anti-E2F1 antibodies at 4°C overnight and with 20 µL Magna ChIP Protein A/G Magnetic Beads (Millipore). Reverse crosslinking was conducted overnight using NaCl at 65°C. The level of GRP78 was determined by qPCR.

### Statistical analysis

All experiments were repeated at least 3 times. Data were analyzed using GraphPad Prism 6.0 and are presented as the mean ± SD. Differences were evaluated using Student’s t-test and ANOVA. Correlation coefficients between RPL5 and E2F1 were analyzed using the Pearson method. Statistical significance was set at p < 0.05.

## Results

In this study, we investigated the potential role of RPL5 in breast cancer and underlying mechanisms. Western blotting was used to analyze ERS-related factors and autophagy-related factors. We found that RPL5 regulated ERS and autophagy of breast cancer cells via regulating E2F1. The study will pvovide a novel insight for the treatment of breast cancer.

### RPL5 was downregulated in breast cancer

**Figure 1.**
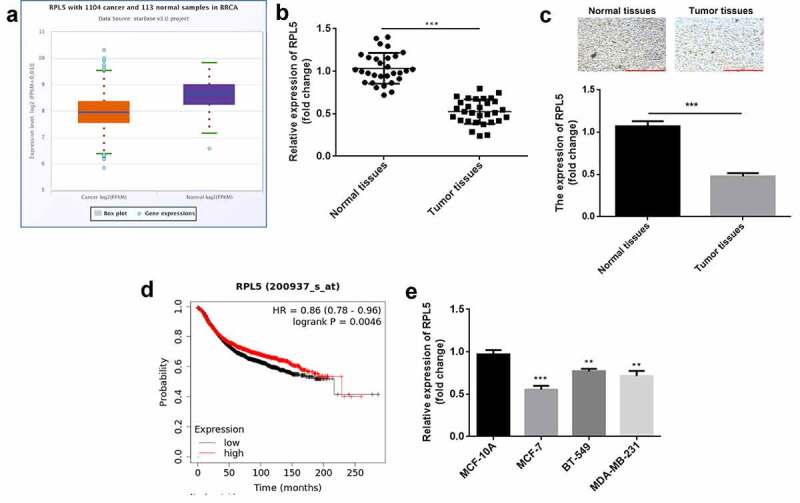
RPL5 was downregulated in breast cancer. (a) RPL5 expression data were acquired from StarBase database. (b) RPL5 mRNA expression was detected in 30 paired tumor tissues and para-carcinoma normal tissues using qPCR. (c) RPL5 expression was detected in tissues using IHC assay. (d) The survival of patients with breast cancer related to RPL5 was acquired from Kaplan online database. (e) RPL5 mRNA expression was evaluated in MCF-7, BT-549, MDA-MB-231 and MCF-10A cells by qPCR. ***P < 0.001. **P < 0.01.

According to the data of StarBase database, RPL5 was downregulated in breast invasive cancer (Figure 1(a)). In this study, the expression of RPL5 was significantly decreased in breast cancer tissues (Figure 1(b)). Similarly, the results of IHC analysis also showed that RPL5 was downregulated in tumor tissues (Figure 1(c)). High RPL5 induced higher survival rate (Figure 1(d)). RPL5 expression was significantly lower in MCF-7, BT-549, and MDA-MB-231 cells than that in MCF-10A cells (Figure 1(e)). MCF-7 cell line was used for subsequent experiments. Moreover, we found RPL5 expression was closely linked to TNM stage, tumor size, and lymph node metastasis, but had little relationship with age (Table 1).

### Overexpression of RPL5 suppressed cell proliferation of breast cancer

**Figure 2.**
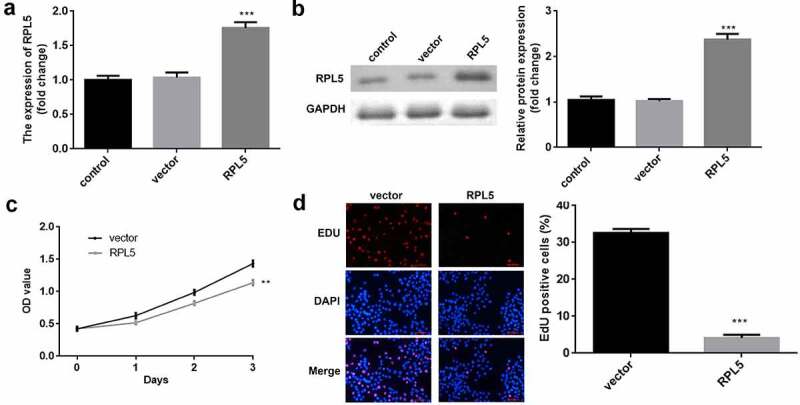
Overexpression of RPL5 suppressed the proliferation of breast cancer cells. (a) Transfection efficiency was estimated for MCF-7 cells transfected with the empty vector and RPL5 overexpressing vector, non-transfected cells were used as the blank control. (b) Transfection efficiency was measured using western blotting. (c) Cell proliferation was determined using CCK-8 assay post-transfection. (d) Cell proliferation was determined after transfection by EdU assay. **P < 0.01. ***P < 0.001.

After transfection, RPL5 expression was significantly upregulated in the ove-RPL5 group, compared with the control and vector groups (Figure 2(a,b)). Furthermore, we found that RPL5 overexpression inhibited the proliferation of breast cancer cells (Figure 2(c,d)).

### RPL5 suppressed ERS-induced autophagy of breast cancer cells

**Figure 3.**
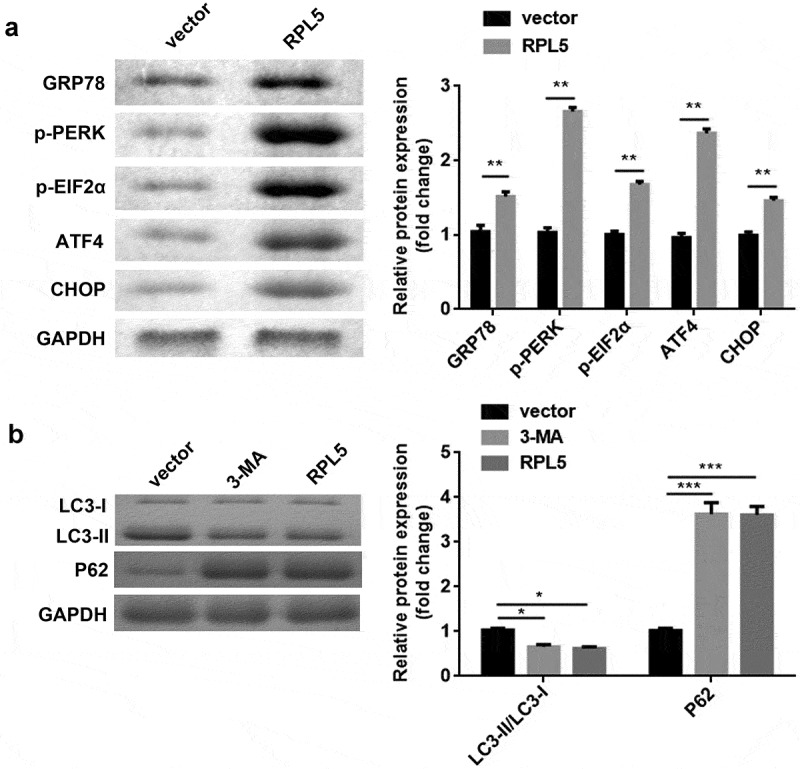
Overexpression of RPL5 regulated ERS and autophagy. (a) The protein expression levels of ERS markers were measured by western blotting. (b) The protein expression levels of autophagy markers were estimated using western blotting. *P < 0.05. **P < 0.01. ***P < 0.001.

After transfection with RPL5 overexpression plasmids, the protein expression levels of ERS markers, including GRP78, p-PERK, p-EIF2α, ATF4, and CHOP, were all remarkably increased (Figure 3(a)). In addition, 3-MA induced the decrease of LC3-II/LC3-I ratio and the increase of P62. Meantime, LC3-II/LC3-I ratio was decreased, and P62 was increased by RPL5 (Figure 3(b)).

### RPL5 downregulated E2F1

**Figure 4.**
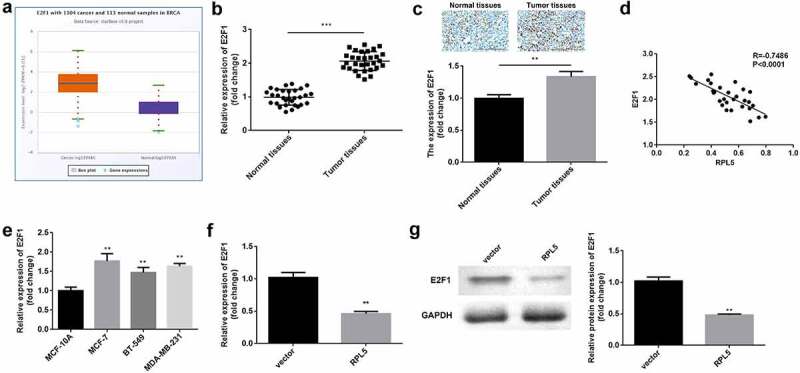
Upregulation of E2F1 was observed in breast cancer. (a) RPL5 expression data were acquired from StarBase database. (b) E2F1 mRNA expression levels were determined in paired tumor tissues and para-carcinoma tissues (n = 30) by qPCR. (c) E2F1 expression was detected in normal and tumor tissues using IHC assay. (d) The correlation between E2F1 and RPL5. (e) E2F1 mRNA expression levels were determined using qPCR in MCF-10A, MCF-7, BT-549, and MDA-MB-231 cells. (f) The mRNA expression levels of E2F1 after overexpressing RPL5 were estimated by qPCR. (g) The protein levels of E2F1 were measured by western blotting. **P < 0.01. ***P < 0.001.

The data from Starbase illustrated that E2F1 was highly expressed in breast invasive cancer (Figure 4(a)). The results of qPCR and ICH assay showed that E2F1 was overexpressed in the tumor tissues (Figure 4(b,c)). E2F1 expression was negatively correlated with RPL5 expression (Figure 4(d)). Additionally, E2F1 was highly expressed in MCF-7, BT-549, and MDA-MB-231 cells, compared with MCF-10A cells (Figure 4(e)). MCF-7 cells were used in the subsequent study. The RPL5 overexpression led to a significant reduction in the expression of E2F1 at both mRNA and protein levels (Figure 4(f,g)).

### Transcriptional activation of ERS marker GRP78 was promoted by E2F1 knockdown

**Figure 5.**
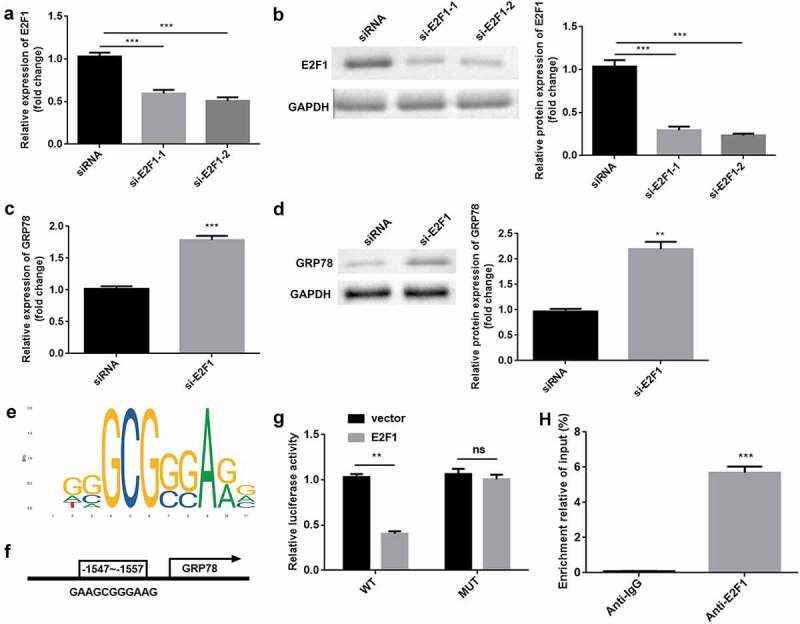
E2F1 transcriptionally inactivates GRP78. (a) The mRNA expression of E2F1 was evaluated in cells transfected with si-E2F1 and siRNA. (b) The protein expression of E2F1 was also evaluated. (c) The mRNA expression of GRP78 was evaluated after E2F1 knockdown. (d) The protein expression of GRP78 was evaluated after E2F1 knockdown. (e) JASPAR online tool was used to predict DNA-binding motifs. (f) The binding sites between E2F1 and the GRP78 promoter. (g) The interaction between E2F1 and the GRP78 promoter was confirmed using a dual-luciferase reporter assay. (h) The reliability of E2F1 binding to the GRP78 promoter was confirmed by CHIP assay. **P < 0.01. ***P < 0.001.

E2F1 was significantly downregulated by si-E2F1-1 and si-E2F1-2 (Figure 5(a,b)). si-E2F1-2 was used in subsequent experiments. E2F1 knockdown induced the upregulation of GRP78 at mRNA and protein levels (Figure 5(c,d)). As illustrated in Figure 5(e), the DNA-binding motif of E2F1 was predicted. Figure 5(f) shows the DNA-binding motif of E2F1 on the GRP78 promoter, which was further verified by luciferase and ChIP assays (Figure 5(g,h)).

### Knockdown of E2F1 regulated ERS and autophagy in breast cancer

**Figure 6.**
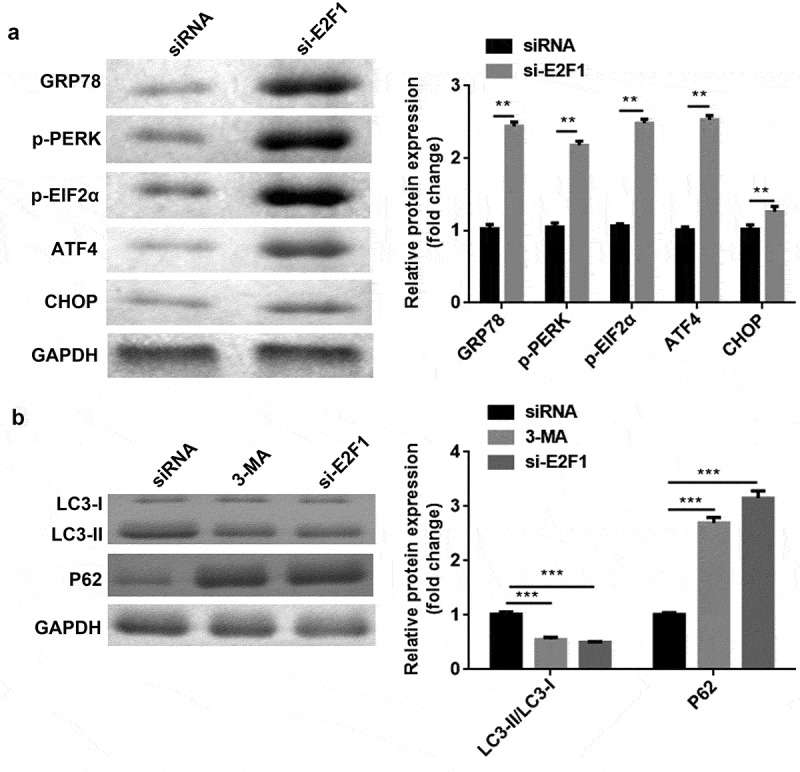
E2F1 knockdown modulated ERS and autophagy. (a) Protein levels of ERS markers were measured by western blotting. (b) The expression levels of autophagy markers were measured using western blotting. **P < 0.01. ***P < 0.001.

In TM-treated cells, E2F1 knockdown enhanced the protein levels of GRP78, p-PERK, p-EIF2α, ATF4, and CHOP (Figure 6(a)). Moreover, 3-MA or E2F1 knockdown upregulated P62 expression but downregulated the ratio of LC3-II to LC3-I (Figure 6(b)).

### Overexpression of RPL5 modulated ERS and autophagy by regulating E2F1

**Figure 7.**
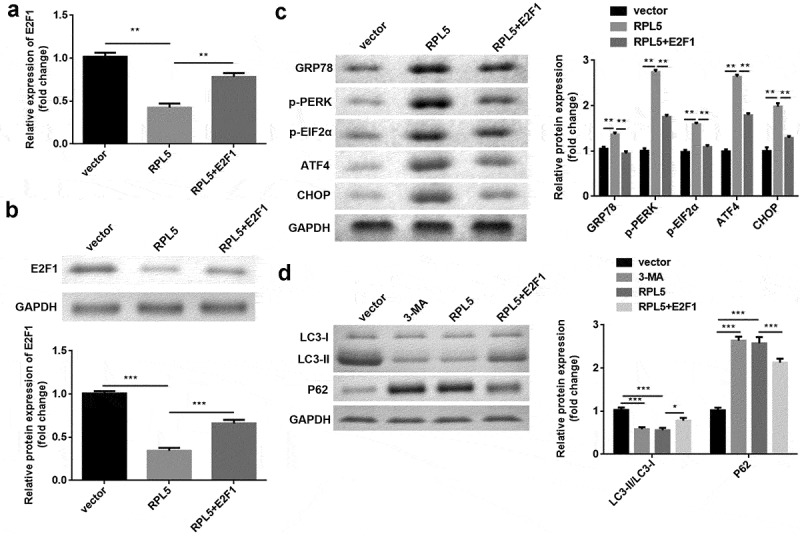
RPL7/E2F1 axis regulated ERS and autophagy in breast cancer cells. (a) The expression of E2F1 was evaluated by qPCR. (b) The expression of E2F1 was evaluated by western blotting. (c) Protein levels of ERS markers were measured by western blotting. (d) Protein levels of autophagy markers were measured using western blotting. **P < 0.01. ***P < 0.001.

After transfection, E2F1 expression was significantly reduced by RPL5 overexpression, which was rescued by E2F1 overexpression (Figure 7(a,b)). Furthermore, the protein expression levels of GRP78, p-PERK, p-EIF2α, ATF4, and CHOP were significantly increased by RPL5 overexpression, whereas E2F1 reversed this increase (Figure 7(c)). 3-MA or RPL5 significantly reduced LC3-II/LC3-I ratio and elevated P62 expression, whereas E2F1 overexpression reversed the effects induced by RPL5 (Figure 7(d)).

## Discussion

In the present study, we explored the role of RPL5 in breast cancer. We found that RPL5 inhibited tumor cell growth and autophagy. Moreover, RPL5 promoted ERS and suppressed autophagy by regulating E2F1.

Mutations in RPs have been found to play a role in cancer development [14]. The role of RPL5 has been identified in several cancers. RPL5 acts as a tumor suppressor in glioblastoma, melanoma, and breast cancer [17]. In breast cancer, MeCP2 knockdown suppresses cell proliferation and induces apoptosis by regulating RPL5 transcription [21]. A high RPL5 predicts a good prognosis for patients with breast cancer [22]. The present study showed that RPL5 is downregulated in breast cancer, whereas its overexpression inhibits tumor cell growth. These results suggest that RPL5 functions as an antitumor gene in breast cancer, which is consistent with previous studies [17,21].

Under homeostatic conditions, PERK binds GRP78 in an inactive state. Upregulation of GRP78 is involved in cancer progression [9,23]. Misfolded proteins accumulate in the ER, leading to ERS, which activates UPR. The activation of UPR regulates tumor cellular processes such as cell growth, autophagy, metastases, and angiogenesis [24]. PERK is a target of the UPR pathway. When ERS is activated, PERK can trans-autophosphorylate and then induce phosphorylation of eIF2α, which reduces protein translation and folding [25]. Furthermore, PERK activates ATF4, which is involved in protein folding, autophagy, and metabolism, to inhibit protein translation [26]. PERK hyperactivation increases the expression of the transcription factor CHOP, inhibiting the anti-apoptotic factor BCL2, which accelerates cell death [27]. Thus, targeting the UPR may be an effective strategy to treat cancer. Accumulating evidence has demonstrated that ERS is associated with autophagy. Autophagy is a part of the ERS response [28]. Induction of autophagy reduces ERS by removing misfolded and aggregated proteins, whereas blocking autophagy increases ERS. Increased ERS and inhibition of autophagy prioritize killing breast cancer cells [29]. During autophagy, a cytosolic form of LC3 (LC3-I) is conjugated to phosphatidylethanolamine to form LC3-phosphatidylethanolamine conjugate (LC3-II), LC3-II/LC3-I ratio increases [30]. In this study, the overexpression of RPL5 induced ERS in breast cancer cells. Moreover, overexpression of RPL5 enhanced the P62 level and reduced the LC3-II/LC3-I ratio, suggesting that RPL5 promotes ERS and suppresses autophagy in breast cancer cells.

E2F1, which belongs to the E2F family, is a transcription factor that transcriptionally induces adenoviral E2. It is the most important protein for cells to enter the S phase and is involved in cell apoptosis, DNA damage response, metabolism, and chemotherapeutic resistance [^31–33^]. High E2F1 expression is found in breast cancer tissues and is associated with a poor prognosis [34]. E2F1 promotes tumor cell viability, metastasis, and cell cycle in breast cancer cells [35,36]. Moreover, E2F1 is associated with autophagy. A previous study reported that the activation of E2F1 enhances autophagy by increasing LC3, ATG1, ATG5, and DRAM expression, but the inhibition of E2F1 suppresses DNA damage-induced autophagy [37]. Additionally, E2F1 plays a crucial role in ERS. E2F1 decline is a late event during the ER response, which inhibits GRP78 at the transcriptional level, and its deficiency promotes eIF2α phosphorylation [38,39]. Inhibition of E2F1 increases the sensitivity of cells to ERS-induced apoptosis [40]. However, the effects of E2F1 on ERS-induced autophagy remain unclear. This study revealed that E2F1 is weakly expressed in breast cancer tissues. E2F1 knockdown promotes ERS and suppresses autophagy. These results suggest that E2F1 serves as a tumor promoter. Furthermore, E2F1 reversed the effects of RPL5 on ERS and autophagy, suggesting that RPL5/E2F1 modulates ERS and autophagy in breast cancer.

## Conclusion

RPL5 was downregulated and E2F1 was upregulated in breast cancer. Overexpression of RPL5 inhibited tumor cell growth and knockdown of E2F1 transcriptionally activated GRP78. Moreover, RPL5 modulated ERS and autophagy by regulating E2F1 expression. This study suggests that RPL5 might be a potential target for breast cancer therapy.

## Data Availability

The datasets used and analyzed during the current study are available from the corresponding author on reasonable request.
